# Risk factors for perinatal mortality in Murmansk County, Russia: a registry-based study

**DOI:** 10.1080/16549716.2017.1270536

**Published:** 2017-01-27

**Authors:** Anna A. Usynina, Andrej M. Grjibovski, Alexandra Krettek, Jon Øyvind Odland, Alexander V. Kudryavtsev, Erik Eik Anda

**Affiliations:** ^a^ Department of Community Medicine, Faculty of Health Sciences, UiT The Arctic University of Norway, Tromsø, Norway; ^b^ International School of Public Health, Northern State Medical University, Arkhangelsk, Russia; ^c^ Department of Preventive Medicine, International Kazakh-Turkish University, Turkestan, Kazakhstan; ^d^ Department of International Public Health, Norwegian Institute of Public Health, Oslo, Norway; ^e^ Department of Public Health, Hygiene and Bioethics, Institute of Medicine, North-Eastern Federal University, Yakutsk, Russia; ^f^ Department of Biomedicine and Public Health, School of Health and Education, University of Skövde, Skövde, Sweden; ^g^ Department of Internal Medicine and Clinical Nutrition, Institute of Medicine, Sahlgrenska Academy at University of Gothenburg, Gothenburg, Sweden; ^h^ Department of Public Health, Faculty of Health Sciences, University of Pretoria, Pretoria, South Africa

**Keywords:** Birth registry, Northwest Russia, perinatal death

## Abstract

**Background:** Factors contributing to perinatal mortality (PM) in Northwest Russia remain unclear. This study investigated possible associations between selected maternal and fetal characteristics and PM based on data from the population-based Murmansk County Birth Registry.

**Objective:** This study investigated possible associations between selected maternal and fetal characteristics and PM based on data from the population-based Murmansk County Birth Registry.

**Methods**: The study population consisted of all live- and stillbirths registered in the Murmansk County Birth Registry during 2006–2011 (n = 52,806). We excluded multiple births, births prior to 22 and after 45 completed weeks of gestation, infants with congenital malformations, and births with missing information regarding gestational age (a total of n = 3,666) and/or the studied characteristics (n = 2,356). Possible associations between maternal socio-demographic and lifestyle characteristics, maternal pre-pregnancy characteristics, pregnancy characteristics, and PM were studied by multivariable logistic regression. Crude and adjusted odds ratios with 95% confidence intervals were calculated.

**Results**: Of the 49,140 births eligible for prevalence analysis, 338 were identified as perinatal deaths (6.9 per 1,000 births). After adjustment for other factors, maternal low education level, prior preterm delivery, spontaneous or induced abortions, antepartum hemorrhage, antenatally detected or suspected fetal growth retardation, and alcohol abuse during pregnancy all significantly increased the risk of PM. We observed a higher risk of PM in unmarried women, as well as overweight or obese mothers. Maternal underweight reduced the risk of PM.

**Conclusions**: Our results suggest that both social and medical factors are important correlates of perinatal mortality in Northwest Russia.

## Background

Perinatal mortality (PM) is an important indicator of the health status of a population. Globally, 6.3 million perinatal deaths occur annually, with considerable variation in these numbers between countries [1]. International comparisons are challenging as countries apply different definitions of PM. In 2000, PM ranged from 111 per 1,000 births in Mauritania to 4 per 1,000 births in the Czech Republic and in Singapore [1]; this difference partly reflects real differences in PM but is also influenced by the different definitions that are used [2].

Before 2012, PM in Russia was defined as death from 28 completed weeks of gestation to 7 completed days after delivery. By this definition, PM in Russia has gradually decreased from 17.9 per 1,000 births in 1990 to 7.4 per 1,000 births in 2010 [3]. A hospital-based registry study in Northwest Russia demonstrated a decrease in PM from 38.2 per 1,000 births in 1987 to 5.4 per 1,000 births in 1996 [4]. In 2012, Russia adopted the World Health Organization (WHO) definition of PM; that is, the number of deaths of fetuses weighing ≥ 500 g (or born at 22 completed weeks of gestation with unknown birthweight [BW]) and newborns up to 7 completed days after delivery, per 1,000 births [5]. National statistics since 2012, therefore, report all stillbirths from 22 weeks of gestation and early neonatal deaths (babies born alive that died within 7 postnatal days). After adopting the WHO definition, PM in Russia increased from 7.2 per 1,000 births in 2011 to 10.0 per 1,000 births in 2012 [3]. Available data exhibit a downward trend in PM in Murmansk County with a decrease from 8.8 [6] to 6.3 [7] per 1,000 births in 2005 and 2011, respectively.

Socio-demographic factors, general health status, as well as availability and quality of medical care associate with pregnancy outcomes, but the impact of these factors on PM varies within and between countries [1]. Unmarried women, as well as women with lower levels of education, exhibit a greater risk of poor pregnancy outcomes [8,9]. The association between advanced maternal age and the risk of PM remains uncertain; some studies suggest that PM increases with maternal age [10,11], while others do not report such an association [12]. Furthermore, cigarette smoking and excessive alcohol consumption associate with stillbirth [13,14]; and obesity, hypertension, and preexisting diabetes mellitus types 1 and 2 are established risk factors for PM [15–17]. Antepartum bleeding of unknown origin increases the risk of PM [18]. Fetal growth retardation (FGR) is associated with higher risk of PM [19,20]. Moreover, outcomes of prior pregnancies may influence the outcome of the index pregnancy; for example, a history of a stillbirth increases the risk of stillbirth [21].

Despite a large number of studies on the determinants of PM in high-, low-, and middle-income countries [9,22–28], the evidence from Russia is limited. Additionally, the contribution of different risk factors to PM in Northwest Russia remains unclear. Therefore, the aim of this study was to investigate possible associations between selected risk factors and PM using data from the first Russian birth registry – the Murmansk County Birth Registry (MCBR).

## Methods

### Study design and data source

We conducted a registry-based study with data from the population-based MCBR. Murmansk County is located in Northwest Russia and had a population of 766,281 in 2015 [29]. The MCBR includes data on all live- and stillbirths from 22 weeks of gestation in Murmansk County from the initiation of the registry on 1 January 2006. The coverage is 98.9% [30]. A standardized form is completed for every birth and contains information on socio-demographic and lifestyle characteristics, reproductive history, pregnancy complications, and characteristics of delivery and the early neonatal period [28,30].

### Study population

**Figure 1.**
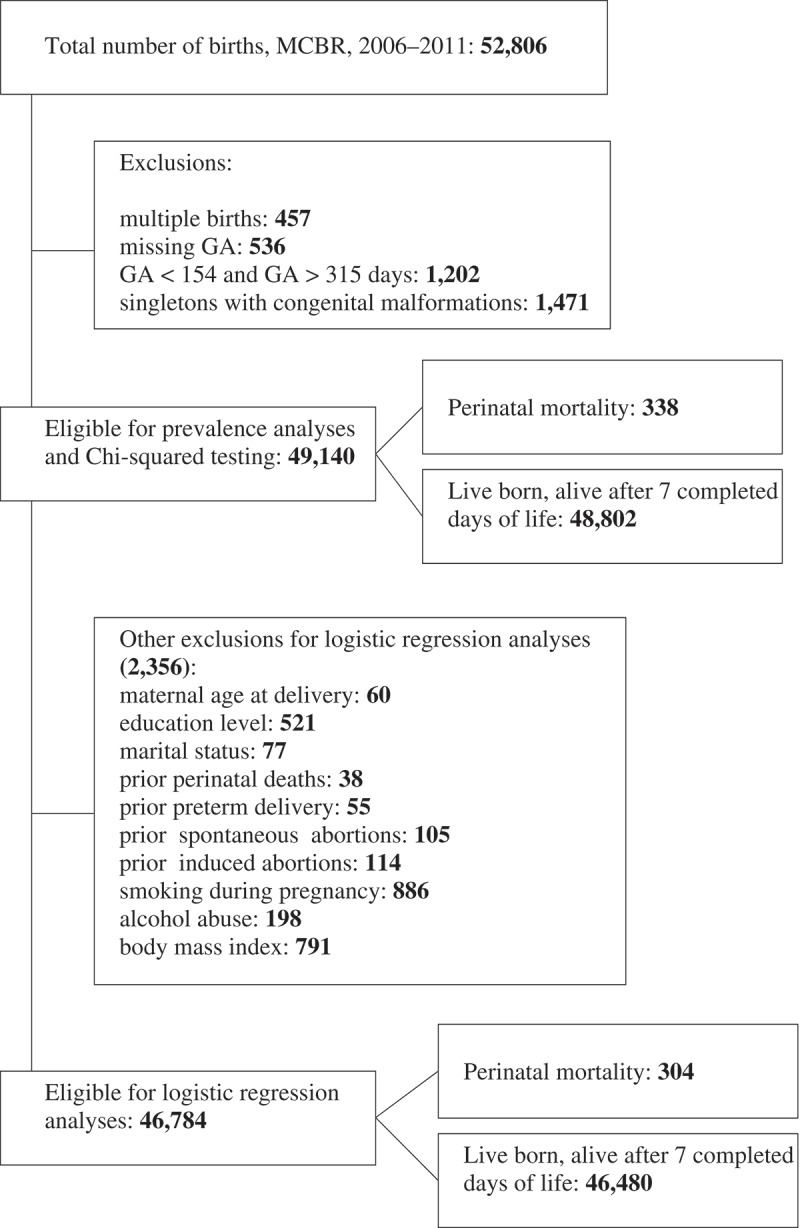
Flow chart of the sampling procedure. Notes: The figure shows the number of births recorded in the Murmansk County Birth Registry in 2006–2011 and the number of births found eligible for this study. MCBR: Murmansk County Birth Registry, GA: gestational age.

The study population consisted of all live- and stillbirths registered in the MCBR between 1 January 2006 and 31 December 2011 (n = 52,806). We excluded multiple births (n = 457) and births prior to 22 and after 45 completed weeks (< 154 and > 315 days) of gestation (n = 1,202) (Figure 1). To investigate potentially preventable risk factors, we applied an approach that has been earlier used in other studies [19,31–33] and therefore excluded infants with congenital malformations (n = 1,471). Gestational age (GA) was determined based on the last menstrual period (LMP). If LMP was missing (n = 1,251), we calculated GA based on first ultrasound. Women with missing information on both LMP and first ultrasound (n = 536) were excluded from the study. Altogether 49,140 births were eligible for prevalence analyses. Those births with missing information on the characteristics under investigation (n = 2,356) were excluded from further risk factor analyses (Figure 1).

### Measurement of variables

We used the WHO definition of PM, i.e. all deaths occurring from 22 weeks of gestation to 7 completed days after delivery per 1,000 births [5]. Data were collected from the MCBR on socio-demographic and lifestyle characteristics, maternal pre-pregnancy characteristics, and maternal pregnancy characteristics. Maternal socio-demographic characteristics included maternal age at the time of delivery (< 18, 18–34, ≥ 35 years), maternal education level (none/primary, secondary, vocational or technical school, and university), marital status (married, cohabiting, and single which included divorced/separated), smoking during pregnancy, and evidence of alcohol abuse during pregnancy (ICD [International Classification of Diseases] 10 code F10).

Maternal pre-pregnancy characteristics included parity, prior perinatal death, prior preterm delivery (i.e. occurring before 37 completed weeks of gestation), prior spontaneous abortions (from 0–22 weeks), induced abortions, and presence of pre-gestational diabetes mellitus type 1 or 2. Previous perinatal deaths, previous preterm deliveries, and previous spontaneous and induced abortions were entered as dichotomous variables (yes, no). Pre-pregnancy diabetes mellitus type 1 and 2 were combined into one dichotomous variable.

Maternal pregnancy characteristics included several dichotomous variables: antepartum hemorrhage, preeclampsia/eclampsia, excessive weight gain (ICD 10 code O26.0), and antenatally detected or suspected FGR (ICD 10 code O 36.5). Early-pregnancy body mass index (BMI) was calculated at the first antenatal visit as the ratio between weight in kg and height in m^2^ (underweight: < 18.5 kg/m^2^, normal weight: 18.5–24.9 kg/m^2^, overweight and obese: ≥ 25.0 kg/m^2^).

### Statistical analysis

We used binary logistic regression to estimate associations between the aforementioned variables and PM. We analyzed the data as cross-sectional as no additional measurements were done over time. Reference categories were taken from previously published studies [8,9]. We also performed Chi-squared tests to evaluate differences in prevalence of studied factors between the PM group and group without PM. Only variables associated with the outcome at *p* ≤ 0.2 were included in the model in the multivariable analysis with the enter method of data entry. We examined interactions between maternal smoking and antenatally detected/suspected FGR, between alcohol abuse and antenatally detected/suspected FGR, as well as between smoking and alcohol consumption. No significant interactions were found between the studied explanatory variables. We calculated crude and adjusted odds ratios (ORs) with 95% confidence intervals (CI). Given a low prevalence of the outcome, ORs calculated by logistic regression can serve as proxy estimates of relative risks. All statistical analyses were performed using SPSS software, v.23.0 (IBM Corp., 2015).

## Results

Overall PM was 8.8 per 1,000 births, which means that 466 deaths occurred among the study population (n = 52,806). After the exclusion criteria were applied, there were 338 perinatal deaths among the 49,140 births eligible for prevalence analysis, yielding a PM of 6.9 per 1,000 births.

Bivariate associations between selected maternal and fetal characteristics and PM are presented in Tables 1–3.

**Table 1.** Bivariate analyses of maternal socio-demographic characteristics as risk factors for perinatal mortality, MCBR, Russia, 2006–2011.

Characteristics	Non-cases N = 48,802		Cases of perinatal mortality N = 338		Crude OR	95% CI	*p*-value*
	N	%	N	%			
Age at delivery, years							0.001
< 18	682	1.4	9	2.7	2.05	1.05–4.00	
18–34	43,771	89.8	282	83.7	1.0		
≥ 35	4,290	8.8	46	13.6	1.66	1.22–2.28	
Education level							< 0.001
None or primary	1,618	3.4	24	7.2	2.74	1.74–4.32	
Secondary	15,278	31.6	129	38.7	1.56	1.19–2.05	
Vocational	15,312	31.7	93	27.9	1.12	0.84–1.51	
University	16,078	33.3	87	26.1	1.0		
Marital status							< 0.001
Single	4,728	9.7	49	14.5	1.84	1.35–2.52	
Married	35,770	73.4	201	59.5	1.0		
Cohabiting	8,227	16.9	88	26.0	1.90	1.48–2.45	
Smoking during pregnancy							< 0.001
No	39,125	81.6	242	73.8	1.0		
Yes	8,801	18.4	86	26.2	1.58	1.23–2.03	
Evidence of alcohol abuse^a^							< 0.001
No	48,441	99.7	330	98.5	1.0		
Yes	166	0.3	5	1.5	4.42	1.80–10.83	

Notes: ^a^ICD 10 code F10.

*The *p*-value refers to comparison of proportions between the PM group and the group without PM for each studied characteristic.

MCBR: Murmansk County Birth Registry; N: number; CI: confidence interval; OR: odds ratio.

**Table 2.** Bivariate analyses of maternal pre-pregnancy characteristics as risk factors for perinatal mortality, MCBR, Russia, 2006–2011.

Characteristics	Non-cases N = 48,802		Cases of perinatal mortality N = 338		Crude OR	95% CI	*p*-value*
	N	%	N	%			
Parity							0.732
Primiparous women	26,858	55.1	183	54.1	1.0		
Para	21,912	44.9	155	45.9	1.04	0.84–1.29	
Prior perinatal death							0.058
No	48,166	98.8	330	97.6	1.0		
Yes	598	1.2	8	2.4	1.95	0.96–3.96	
Prior preterm delivery^a^							< 0.001
No	47,711	97.9	317	94.1	1.0		
Yes	1,037	2.1	20	5.9	2.90	1.84–4.58	
Prior spontaneous abortions (0–22 weeks)							0.003
No	42,884	88.1	280	82.8	1.0		
Yes	5,813	11.9	58	17.2	1.53	1.15–2.03	
Prior induced abortions							< 0.001
No	28,093	57.7	163	48.2	1.0		
Yes	20,595	42.3	175	51.8	1.46	1.18–1.81	
Pre-gestational diabetes mellitus type 1 or 2							0.094
No	48,709	99.8	336	99.4	1.0		
Yes	93	0.2	2	0.6	3.12	0.77–12.70	

Notes: ^a^Delivery occurring after the 22nd completed week and before the 37th completed week of gestation.

*The *p*-value refers to comparison of proportions between the PM group and the group without PM for each studied characteristic.

MCBR: Murmansk County Birth Registry; N: number; CI: confidence interval; OR: odds ratio.

**Table 3.** Bivariate analyses of maternal pregnancy characteristics as risk factors for perinatal mortality, MCBR, Russia, 2006–2011.

Characteristics	Non-cases N = 48,802		Cases of perinatal mortality N = 338		Crude OR	95% CI	*p*-value*
	N	%	N	%			
Antepartum hemorrhage							0.034
No	47,428	97.2	322	95.3	1.0		
Yes	1,374	2.8	16	4.7	1.72	1.04–2.84	
Preeclampsia/eclampsia							0.469
No	44,580	91.3	305	90.2	1.0		
Yes	4,222	8.7	33	9.8	1.14	0.80–1.64	
Excessive weight gain^a^							0.076
No	44,929	92.1	320	94.7	1.0		
Yes	3,873	7.9	18	5.3	0.65	0.41–1.05	
Early pregnancy BMI, kg/m^2^							0.001
Normal weight (18.5–24.9)	31,590	65.8	195	61.7	1.0		
Underweight (< 18.5)	3,064	6.4	8	2.5	0.42	0.21–0.86	
Overweight and obese (≥ 25.0)	13,379	27.8	113	35.8	1.37	1.08–1.73	
Antenatally detected/suspected FGR^b^							< 0.001
No	47,416	97.2	313	92.6	1.0		
Yes	1,386	2.8	25	7.4	2.73	1.81–4.12	

Notes: ^a^ICD 10 code O26.0.

^b^ICD 10 code 036.5.

*The *p*-value refers to comparison of proportions between the PM group and the group without PM for each studied characteristic.

MCBR: Murmansk County Birth Registry; N: number; CI: confidence interval; OR: odds ratio; BMI: body mass index; FGR: fetal growth retardation.

There were 86.6% term and 5.9% post-term babies among the 49,140 births eligible for prevalence analyses. Extremely preterm (< 28 weeks), very preterm (28–31 weeks), and moderate-to-late preterm (32–36 weeks) infants accounted for 1.0, 0.4, and 6.1%, respectively. In our study population, the highest proportion of infants (85.1%) was born with BW 2,500–3,999 g. Extremely low BW (< 1000 g), very low BW (1,000–1,499 g), and low BW (1,500–2,499 g) infants accounted for 0.4, 0.4, and 3.8%, respectively. The observed prevalence of heavy babies (4,000 g and more) was 10.3%. The highest proportion of PM (36.7%) was in infants born at GA 22–27 weeks. The distribution of PM in infants born at GA 28–31, 32–36, 37–41, and 42+ weeks was 4.7, 21.6, 33.4, and 3.6%, respectively.

Mean BW and standard deviation (SD) in the group without PM were 3,383 (513) g. In the PM group, mean BW (SD) comprised 1,958 (1,164) g. Median GA with interquartile range for the groups with and without PM were equal to 238 (192–275) and 279 (272–285) days, respectively.

### Mothers’ socio-demographic characteristics

Mothers with the lowest education level were more likely to experience PM compared to those with higher education (Table 1). In bivariate analysis, the risk of PM was 66% higher in women aged ≥ 35 years compared to those aged 18–34 years; single and cohabiting women exhibited increased risk of PM compared to married women. Smokers and those with evidence of alcohol abuse during pregnancy were also at higher risk of PM. After controlling for other characteristics in multivariable analysis, single and cohabiting mothers, women with evidence of alcohol abuse, as well as mothers with the lowest education continued to display a higher risk of PM (Table 4).

**Table 4.** Multivariable analysis of risk factors for perinatal mortality, MCBR, Russia, 2006–2011.

Characteristics	Adjusted OR*	95% CI
Socio-demographic characteristics		
Age at delivery, years		
< 18	1.32	0.61–2.85
18–34	1.0	
≥ 35	1.22	0.85–1.75
Education		
None or primary	1.98	1.17–3.36
Secondary	1.18	0.87–1.59
Vocational	0.90	0.67–1.23
University	1.0	
Marital status		
Single	1.54	1.08–2.20
Married	1.0	
Cohabiting	1.72	1.31–2.27
Smoking during pregnancy		
No	1.0	
Yes	1.10	0.83–1.45
Evidence of alcohol abuse		
No	1.0	
Yes	2.94	1.05–8.27
Maternal pre-pregnancy characteristics		
Prior perinatal death		
No	1.0	
Yes	1.12	0.49–2.54
Prior preterm delivery		
No	1.0	
Yes	2.16	1.25–3.71
Prior spontaneous abortions (0–22 weeks)		
No	1.0	
Yes	1.41	1.04–1.91
Prior induced abortions		
No	1.0	
Yes	1.35	1.07–1.71
Pre-gestational diabetes mellitus type 1 or 2		
No	1.0	
Yes	3.25	0.79–13.41
Maternal pregnancy characteristics		
Antepartum hemorrhage		
No	1.0	
Yes	1.89	1.13–3.14
Excessive weight gain		
No	1.0	
Yes	0.72	0.44–1.15
Early pregnancy BMI, kg/m^2^		
Normal weight (18.5–24.9)	1.0	
Underweight (< 18.5)	0.44	0.21–0.89
Overweight and obese (≥ 25.0)	1.31	1.03–1.67
Antenatally detected/suspected FGR		
No	1.0	
Yes	2.57	1.66–3.97

Notes: MCBR: Murmansk County Birth Registry; CI: confidence interval; OR: odds ratio; BMI: body mass index; FGR: fetal growth retardation.

*Adjusted for all other variables in the model.

### Maternal pre-pregnancy characteristics

Bivariate analysis of maternal pre-pregnancy characteristics showed that prior preterm delivery and prior spontaneous and induced abortions associated with increased risk of PM (Table 2).

Prior preterm delivery, and prior spontaneous or induced abortions increased the risk of PM after adjustment for other studied maternal characteristics in multivariable analysis (Table 4).

### Maternal pregnancy characteristics

Bivariate analyses showed that women with antepartum hemorrhage and overweight/obese women were at higher risk of PM (Table 3). Antenatally detected/suspected FGR demonstrated 2.7-fold increased risk of PM. Preeclampsia/eclampsia contributed to a non-significant increased risk of PM. Underweight women had lower risk of PM. In the multivariable analysis, antepartum hemorrhage and overweight/obesity remained significantly associated with PM after adjustment for all other socio-demographic, pre-pregnancy, and pregnancy characteristics. Overweight and obese mothers had a 30% higher risk of PM compared to normal-weight women (Table 4). Underweight women continued to exhibit reduced risk of PM in the final model. Antenatally detected/suspected FGR was associated with 2.6-fold increased risk of PM.

### Infant birth weight

One hundred and forty four perinatal deaths (42.9%) occurred in infants having BW of < 1,500 g. The smallest proportion (3.3%) in the PM group was among infants with a BW of > 4,000 g. In bivariate analyses, fetuses and newborns with BW of 1,500–2,499 g had almost a 17-fold risk of PM compared with those having BW of 2,500–4,000 g. Heavy infants did not exhibit increased risk of PM in our study.

### Perinatal deaths in births excluded from the study

Among excluded singleton births due to missing or inapplicable GA, or congenital malformations (a total of 3,209 births) (Figure 1), 81 (2.5%) infants died during the perinatal period compared to 338 babies (0.7%) from women without missing data. PM in 1,471 infants with congenital malformations was 21.1 per 1,000 births (31 cases of PM). It was almost three-fold higher compared to PM in our study sample. Mothers in the group with missing/not applicable data were, compared to those without missing data, more likely to be < 18 years old (3.3 vs 1.4%, *p *< 0.001) at the time of delivery. A higher proportion of women smoked during their current pregnancy (26.3 vs 18.4%, *p *< 0.001), had the lowest level of education (7.0 vs 3.4%, *p *< 0.001), abused alcohol (2.7 vs 0.3%, *p *< 0.001), and were single (16.7 vs 9.7%, *p *< 0.001).

There were 47 perinatal deaths among 918 infants born by 457 women pregnant with multiples. Together, this accounted for a PM of 51.2 per 1,000 multiple births. Babies from multiple pregnancies contributed 10.1% of all PM. Twenty seven deaths occurred among second infants, 25 babies (53.2% of PM in multiple births) were stillborn.

## Discussion

We found that PM in Murmansk County was 8.8 per 1,000. After applying exclusion criteria (multiple births, infants with congenital malformations, and births with missing GA and GA prior to 22 and after 45 completed weeks), PM in Murmansk County was reduced to 6.9 per 1,000. Both figures are lower than the national Russian average of 9.6 reported in 2006 [34] as well as PM in a previous study based on MCBR data (10.7 per 1,000) [28]. Our data suggesting PM of 6.9 are comparable with a PM of 7.2 as reported in Russia for 2011 [7]. The main reason for lower PM in our study may be that we excluded multiple births, infants with congenital malformations, and records with missing information on GA and/or studied characteristics.

### Socio-demographic characteristics and PM

We identified associations between maternal socio-demographic characteristics and PM with a similar pattern as described earlier [9–11]. We found that maternal education was an independent predictor of PM, which agrees with our earlier study in Northwest Russia [8]. Maternal low education was also a statistically significant predictor of PM even after controlling for other maternal pre-pregnancy and pregnancy characteristics.

The association between advanced maternal age and PM was demonstrated in the bivariate analysis. In our study, advanced maternal age did not correlate with PM after controlling for other maternal and fetal characteristics. Age-related confounding or intermediate factors might have an effect on the association between maternal age and adverse pregnancy outcome [10,11]. Parity, BMI, ethnic origin, and mostly social deprivation are confounders in association with maternal age and stillbirth; the confounding effect of smoking is limited [11]. Hypertension has also been indicated as an intermediate factor in the relation between maternal age and adverse pregnancy outcomes, and low education acts as a confounder [10]. Pregnancy complications not included in this study might be intermediate factors in the association between maternal age and PM as described [10].

Maternal smoking increased risk of PM in the bivariate analysis but lost its statistical significance in the multivariable model. Underreported prevalence of smoking may contribute to these findings. Other studies confirm an association between maternal cigarette smoking and stillbirth [13] or PM [35]. Our findings that infants of alcohol-abusing mothers are at higher risk of PM are in line with results of other studies that demonstrate prenatal alcohol exposure as a predisposing factor for stillbirth [14,36].

### Maternal pre-pregnancy characteristics and PM

The contribution of prior adverse pregnancy outcome to PM has been described [37]. In our study, maternal pre-pregnancy characteristics increased the risk of PM when the woman had a history of preterm delivery or spontaneous and induced abortions. Prior preterm delivery and prior induced and spontaneous abortions were the significant risk factors associated with PM after adjustment for other socio-demographic, pre-pregnancy, and pregnancy characteristics. Previous preterm delivery is a strong predictor of future preterm births [38], and preterm birth and PM are associated [1,39]. Stillbirth during the first pregnancy associates with higher risk of stillbirth also happening during the second pregnancy. After adjustment for confounders, mothers with a previous stillbirth exhibit an almost two-fold risk of stillbirth in their next pregnancy compared to mothers with live births [21].

The association of parity and PM is not clear. Some studies demonstrate decreased risk of PM in para women [23,40], others indicate that high parity promotes higher risk of obstetric complications which then increase the risk of PM [41]. However, we found no significant difference in PM between primipara and para women. This might be explained by the young age of our study population and the fact that these women seldom had more than two children. Previous studies show that parity modifies the effect of maternal age on adverse pregnancy outcomes [24,42]. The effect of maternal age is strong for the first birth, but does not influence subsequent births [24].

### Maternal pregnancy characteristics and PM

Preeclampsia and eclampsia during pregnancy are risk factors for PM [25]. In our study, preeclampsia/eclampsia showed a trend towards contributing to an increased risk of PM but this result was not statistically significant. One reason might be a different approach to registering such pregnancy complications in maternity wards in Murmansk County. Indeed, the qualifications of the medical personnel and diagnostic capabilities may pose validity problems in birth registries [9].

We found that antepartum hemorrhage was significantly associated with PM. Other studies demonstrate an independent association between antepartum hemorrhage and stillbirth as well as early neonatal death [22,43]. As antepartum hemorrhage contributed to a 1.7-fold increased risk for PM in our study, its role needs to be addressed in future studies.

Furthermore, we found increased risk of PM in babies born by overweight or obese women, which has earlier been described in a meta-analysis exploring maternal obesity and risk of stillbirth [16]. To date, the mechanisms for the association between maternal obesity and PM are still unresolved. Overweight and obesity may exert their effect through placental insufficiency [44] or through other pregnancy complications that are associated with obesity in pregnant women [16].

In our study, infants of underweight women had lower risk of PM compared to normal-weight mothers. It is unclear why this occurs, but could be due to lower age in women having low BMI compared to normal or overweight/obese mothers. The proportion of underweight women (9.5%) was highest among young mothers compared to those aged 18–34 years (1.3%) and > 35 years (6.8%). Other studies demontrate that low maternal BMI does not increase risk of fetal death [44,45].

In our study, antenatally detected/suspected FGR increased risk of PM in both bivariate and multivariable analyses. This pathology contributed to a 2.6-fold increased risk for PM after adjustment for all other variables in the final model. These findings are in line with a recently published study [19] that found 7.8-fold higher risk of stillbirth in non-smoking women who had antenatally detected FGR. Compared to the aforementioned study, our study does not suggest an interaction between maternal smoking and detected or suspected FGR during current pregnancy.

### PM among low birthweight and preterm infants

In our study both mean BW and median GA were lower in the PM group compared to the group without PM. Our finding of BW-specific PM is in line with earlier studies in Murmansk County [28] and the United States [46]. Preterm newborns have higher risk of death during the first week of life [1] as well as neonatal and infant mortality [46]. In 2006, WHO reported that prematurity is responsible for 62% of early neonatal deaths [26].

### Limitations of the study

Limitations of our study include the absence of data on ultrasound-estimated GA, which were not included in the registry before 1 January 2009. We attempted to unify the data for 2006–2011 and calculated GA for all births in the MCBR. LMP and first ultrasound data were used to determine GA as the first trimester report of LMP corresponds to GA based on data of the first trimester ultrasound [47]. However, our approach may limit the accuracy of GA. Furthermore, the frequency of smoking and alcohol consumption among mothers may be underreported due to self-reporting.

Maternal infections of the genitourinary tract are a common cause of PM [27]. Information on infections of the genitourinary tract during pregnancy is recorded in the MCBR from obstetric records. As there was no unified approach in genitourinary tract infections registration, we did not include this variable in our model.

As no data on pre-pregnancy BMI were recorded in the MCBR, we assessed BMI using information from the mother’s first antenatal care visit. Pre-pregnancy BMI is considered preferable, but a recent study shows the value of using early-pregnancy BMI [48]. There is a recommendation to calculate BMI based on accurate early-pregnancy weight and height measurements, and not on self-reported or pre-pregnancy data.

In total, 5.1% of the study population was excluded from prevalence analyses as they had missing and not applicable data and these women had a much higher PM rate than the study population. Both in an earlier study [28] and in the current study, these women were identified as ‘suddenly’ appearing at a hospital to give birth having had no previous contact with the antenatal care system. The proportion of women delivering at home was low, with 98.9% of all births in Murmansk County recorded in the MCBR [30]. Inclusion of women with missing data into this study population would have been ideal and helped strengthen our results further.

In our study, PM in excluded multiple births was almost 15 times higher compared with PM in singletons included in the study. The contribution of multiple pregnancies to increased risk of PM has been demonstrated [2]. Birth-related complications [49] which were not investigated in our study, as well as low BW and congenital anomalies [50], are important risk factors for PM in multiples. Indeed, twins have a three-fold increased risk of intrapartum stillbirth compared to singleton babies [51]. The exclusion of multiple births as well as singletons with congenital anomalies may have contributed to an underreporting of PM in our prevalence analysis.

### Strengths of the study

The major strength of this study is the large study population, ensuring sufficient statistical power to detect effects of less influential factors compared to previous Russian studies [8]. Additionally, our study is based on validated registry data [30]; therefore findings can be generalized to singleton pregnant women at 22–45 weeks of gestation in the entire region. We were able to include data on many potential risk factors and confounders related to PM for better statistical control.

## Conclusions

Risk factors associated with PM were low education level, unmarried status, prior adverse pregnancy outcomes (preterm deliveries and abortions), antepartum hemorrhage, overweight or obesity, antenatally detected/suspected FGR, and alcohol abuse. Low maternal BMI associated with reduced risk of PM.

## Paper context

This study investigated risk factors of fetal and newborn deaths in Northwest Russia. We used data from a regional birth registry. Maternal low education, prior preterm delivery, abortions, antepartum hemorrhage, detected or suspected fetal growth retardation during current pregnancy, and alcohol abuse increased the risk of fetal and newborn death. Similarly, unmarried women as well as overweight or obese mothers were at higher risk. Being underweight decreased the risk.
